# United States Veterinary Medical Association

**Published:** 1891-10

**Authors:** 


The report was received for discussion.

*The President.* We will now have the Secretary's report.

The Secretary read his report as follows :

### SECRETARY'S REPORT.

*Mr. President and Gentlemen:*

The past year has been a very busy one in Association circles and I trust that much of the work done may be found here to-day, in the evidence of numbers and interest shown in this meeting. Hundreds of letters of inquiry have poured into my office and at the close of my work on Monday morning, not one remained unanswered or unrecognized. We gather together to-day with a new element among us in the delegates from no less than ten State and local associations, while our members are numbered among the most active in every association circle in the land. Our association numbers to-day 270 members actively participating in our work, while in addition we have the support and encouragement of some seven in the roll of Honorary membership ; while to day some thirty more have knocked at your door for admission. Surely these are propitious times and signs of healthy growth. During my incumbency as your Secretary, in round numbers one hundred and fifty new members of the profession have sought your favorable consideration. Should this ratio of increase continue during the balance of ten years from 1888 to 1898, your membership will in all probability, reach 1,000, in our country. Much of this newly added power comes from the youth and strength of the future profession of your wonderful country and it places upon the shoulders of those trained in the service a deep responsibility in guiding this great power and moulding its untold strength, in lines and ways of great use, for the up-building of your veterinary structure in America. On every side in State and county and city, are budding forth associations of veterinarians, all training their guns upon this parent association ; looking for the word of command to so direct the powers of their ammunition, in telling directions where it may not be spent on vacant space and so lost forever in the din and smoke that follow in its train. Upon us falls the grave responsibilities of centering our greatest forces upon all questions of a national character, that the more local work should devolve in its chief detail upon the lesser organizations were time and position fit it better to rest and be performed.

In national sanitary work ; in national meat inspection ; in national laws as to contagious and infectious diseases ; the commercial interests of our nation at home and abroad command our salutary support and influence in sustaining our place among the nations of the world, as the great producer of food and food products for the whole earth, national and state laws to better protect our people from disease direct and indirect, should be yearly considered by this association. Sanitation in its wide and inestimable importance should receive at our hands a broad consideration, that public sentiment might better be moulded to yield to our profession, its chief place as sanitarians in all this work the world over. National ethics of which I hear whispering sounds down along the line of our members, should here be given an impetus, that shall award it, its proper status in country, state and city. Veterinary jurisprudence in the hope of better and more justly uniform laws is before you to-day, and I trust it may sound the alarm down along the ranks, that shall carry sufficient force to return to us again in a clearing away of the debris of useless and worthless decisions, that make up the laws to-day that govern the decision of these important points to us, and thus free us from the injustice we suffer and remove us from the unfair criticism, that often make us the laughing stock of our ignorant employers.

Our country so rapidly assuming a first place among the nations as a producer of the finest specimens of every form and kind of domestic animals, should command our attention in enlightening the world on breeding statistics and the relative part thus played in disease, heredity, etc., etc., should be a self assumed task upon our part. In addition our association should be doing some work looking to the avoidance of the importation of diseases heretofore unknown in our breeding districts, and taking fitting recognition in the future of such out-breaks at the fatal one of "Dourine" in Illinois.

Surely, the older nations of the earth are looking to this youthful, unrestrained but always practical nation, to play a more important part in veterinary annals in the world's work in the future, than we have done in the past. From this organization should emerge the plans and directing forces and I hope we shall not be unmindful to-day of the opportunities that are within our grasp. The world's fair but two years hence offers a fitting opportunity for us to step forward into line with all the older veterinary worlds end, I trust this association will take proper and active steps looking to an International meeting at Chicago in 1893. We should be early in the field with our announcements and thus have all future gatherings of veterinarians planning in unison their work that it may better fit in to the work for us to do in 1893.

Sources of veterinary education in our land should ever be watched by us with a zealous eye, and we should be quick to respond in just recognition to every movement upon the part of veterinary educational institutions to increase their power and to broaden their course of instruction. We cannot control our schools, but we can do that which is better in a nation living under a republican form of government, we can mould and fashion public opinion throughout our land, that shall justly measure to each educational source its value and worth and thus strengthen our own place in national work, that shall make us a more useful body in the future, than we have so filled as an ornamental and obstructive body in the past.

These are a few of the thoughts suggesting themselves to me since I have filled your office as secretary, and I take the liberty of incorporating them in my report.

During the past year our association has been represented in every veterinary gathering held in our country, through the faithful support and assistance of our Assistant State Secretaries, by my presence as your representative, or by letter in your behalf. I have given aid and encouraging support in every meeting of veterinarians and urged their union with us in carrying forward the work that is ours to do. Through public press and veterinary journals I have endeavored to strengthen our organization in every way that I could; keeping before them at all times something of interest to awaken them to a keener sense of duty. Through printed matter the output of your publication committee, I have placed in every nook and corner of our broad land some seed to germinate, that I hope in the future will flower to shed fragrance on this body.

I have compiled for your use a list of veterinarians now numbering 1,300, spread over the entire country, their correct addresses and of what colleges they are graduates. I have almost completed a complete file of every member of this organization since its birth, and only the lack of records so imperfectly kept has forbidden me to trace in completion their entire history as a part of the record of this association. As books of reference for future officers of this association, these books are invaluable. The sending forth of at least three statements of their respective indebtedness to the association of each member, has been a valuable reminder to all, and your increased treasury with a ten-fold multiplication of our expenses, attests the value and efficacy of this work.

No member henceforth can be one of our number without a proper record, being on file, showing his qualifications, etc., neither can he be in possession of our certificate of membership, without their being on file in sustaining his claim, his signature to our Constitution and by-laws, a copy of which has been placed in every member's hands, and one to each new one with his application blank.

For this meeting some nine hundred programs, railroad notices, etc., etc., have been sent out, and at this writing less than ten have been returned undelivered. The newspaper press has been in many ways notified of our existence and no opportunity has been lost to bring our organization into that just prominence she so richly deserves.

All this and much more has been performed in your interest and welfare and it has been at a fearful expenditure of time and labor.

The pecuniary compensation is wholly inadequate to assure you of the continuance of this work, unless our good fortune shall drop on one of you whose heart will grow rapt up in the work, as I have and found my mind continually planning. I would, therefore, recommend the consideration of an increased salary as a stimulus to this work and with the hope that some one will after to-day take up and make grander and better the work already done and thanking you all for you warm support and encouragement, I give you notice at this time, that there are no contingencies or train of circumstances that can lead me to accept this onerous position again. I therefore submit for your consideration my final report.

W. HORACE HOSKINS, *Secretary.*

*The Secretary.* My accounts are here for your examination and approval. I beg to say that letters of regret have been received from Prof. McEachran, Prof. Liautard, Drs. Rouif, John S. Meyer, Hunter, Nesbitt, Grange, Morris, Howard, and Schwartzkopff.

*The President.* The next order of business is the report of State Secretaries. Several state secretaries since morning have been dropped from the association. Therefore, California is not represented. There is no representative from Kentucky. Dr. Cary, of South Dakota, is not present. Dr. Walmer, is present, I. believe. *Dr. Walmer.* I have no report to submit. *The President.* Georgia has no representative, having also been dropped. The Secretary will read the report of Dr. Grange, of Michigan. The Secretary read the report.

No report was received from South Carolina, but the assistant state Secretary Dr. B. McInnes, had forwarded three photographs, one of which was a young calf of a few days old suffering from tetanus. It was a very striking picture. The others were of bitches suffering from tetanus, the sequel of the operation of ovariotomy.

*The President.* The next state represented here is that of Maryland—Dr. Clement. *Dr. Clement.* I have not prepared a report, and therefore have none to offer. *The President.* The next state Secretary is Dr. Lowe, of New Jersey, Dr. Lowe submitted a report:

We have a communication from Dr. Frinck, of New Brunswick. The Secretary read the communication.

*The President.* The report from the College Committee which was postponed in the order of reaching it will now be received.

*Dr. Lyford.* I have letters here from four or five Colleges. The different Colleges to which I put the list of questions were the Americain, New York, Ames, Kansas City, Montreal, University of Minnesota, Chicago, Toronto, Pennsylvania and Harvard. Four answers have come. Why we should not have gotten answers from the nearest colleges to me I do not know. The communications from the New York and Toronto Schools were the first to appear, although they were the farthest off. The questions I put to them for answer were first: What would you consider the necessary requirements for standard uniform Veterinary education? Second: What would you be willing to do in the matter to obtain such a standard? The letters were read as follows:

The Iowa School's report was that they were willing to do anything to increase their course, and would do what they could if it was in accordance with the rest of the schools. The State University of Minnesota has a three years course of nine months each. They are just starting in, and they are now building a large hospital. That is one of the reasons why Prof. Schwartzopff is not here.

*The President.* The first report for discussion before the meeting is that of Dr. Peters on Intelligence and Education. The Chair would suggest that in order to expedite and facilitate and make the discussion of these reports more clear, and to avoid the annoying conversation which we sometimes have, of short questions being answered and perhaps useless questions being asked at times, that each member be allowed a time not exceeding ten minutes for a single discussion. Of course if anything is to be added of special nature the privilege can be accorded. Is there any objection to that mode of discussion. *Dr. Miller.* I move that the discussiou be limited to ten minutes and one final response by the writer. The question was put, and the motion agreed to.

*Dr. Clement.* Mr. President, and Gentlemen, I was very much pleased to hear the report of my friend Dr. Peters this morning. and I must say that I am very much impressed with the fearlessness with which he expresses his opinion. I think we should all follow his example at our meetings. I wish to speak only for a moment upon that part of his report which refers to investigations of swine fever and hog-cholera. I wish to say a few words simply because it has been my opportunity for the last three or four years of doing some work in that line myself in association with Prof. Welch at the Johns Hopkins University. I might say in parenthesis that my work in this line has nothing whatever to do with my position as Government Inspector. That it is independent work performed in the laboratory of the above institution. The conclusions which we have arrived at, expressed in a few words, are these, and I must be careful in mentioning them that I do not anticipate what is to come out in the report winter, namely: that there are two diseases of swine in this country. First there is a hog-cholera, and secondly swine-plague. That the two diseases which occur in hogs of this country are described in the reports of Dr. Smith of the Bureau of Animal Industry; that the organism described by Dr. Billings as swine-plague and that described by Dr. Salmon as hog-cholera are identical. That swine-plague does exist and does cause more or less trouble, is in our opinion without a doubt. As to what connection the organism has with the lesions described in the reports of the Bureau is a question on which we might not all agree. Nevertheless the swine-plague organism does cause trouble. The trouble in hogs is as a rule, in our experience, one of mixed infection. We have not had the opportunity of a seeing an outbreak of swine-plague pure and simple. The remarks which Dr. Peters quotes from Dr. Jeffries' experiments it seems to me are not altogether in keeping with the work which is being done at present.

The conclusions which Dr. Jeffries draws are that there is but one disease of hogs, according to the investigations made at that time. We have found that it is very hard to say when swine-plague is present that hog-cholera is absent, from the fact that swine-plague kills in a few hours, while hog-cholera requires some days. If, then, an animal be killed and presents lesions of the intestines such as are generally supposed to be characteristic of hog-cholera the statement must be very carefully considered before it is made, that hog-cholera is not present. We are thrown off our track once or twice during the earlier part of our investigation. We found afterward that hog-cholera did exist in these animals that we thought had swine-plague pure and simple. We found that some of the colonies were different from others and required cultivation and inoculation into animals and produced cholera even if the inoculation from the sick to the well animal and the characteristics of hog-cholera absent kill the animal. I would simply say in general way that from our investigation we have found that Dr. Billings is right in certain other matters.

As to the identity of the American swine-plague with the German swine-plague I am not at liberty to speak at this moment on account of the absence of my Senior associate in the work.

I cannot say too much either, as I said before, because the report will be forthcoming in the course of a few months at most, and I think it will then be found as fair a report as the investigators in the matter are capable of producing, and as said above that all the parties interested in this long continued discussion are right in certain directions.

*Dr. Kilborne.* I wish to answer one point in Dr. Peters remarks this morning, and that is in regard to the credit due for the investigations of hog-cholera. It is insinuated in that report that whereas Dr. Salmon has been receiving the credit for the work has been done, it turns out now that Dr. Smith has been doing that work all along. That, in one sense, is true, but I have been connected with the Bureau for the last five years in its work, and the investigations in swine disease until the past year has been under the immediate supervision and direction of Dr. Salmon himself. The microscopic work is in charge of Dr. Smith, and the field experiments I have had charge of. If any one will take the trouble to read the report of the Bureau of Animal Industry they will find in every one of the reports that it is distinctly stated in the preface or letter of transmittal that Dr. Smith has had charge of the laboratory and I have had charge of the experiment station. Any one who will look at that can easily conclude the assistants who had done the work, and I do not see how they can accuse the chief of the Bureau of playing double in that matter. The work has been directed by him, the experiments were directed by him, and he had reported the result.

*Dr. Clement.* Can we speak twice on the same question?

*The President*, There is no objection.

*Dr. Clement.* I wish to quote a short paragraph from Dr. Jeffries' article in THE JOURNAL OF COMPARATIVE MEDICINE AND VETERINARY ARCHIVES, December 1890:

"One dead, full grown hog was sent to me for bacteriological examination. Unfortunately decomposition was well under way before it came to hand. Autopsy made by Dr. Peters and myself shows skin red and blue in patches and studded with ecchymoses, especially on the ventral surface. Lungs dark red to purple, resistent but not so solid as in lobar pneumonia; cut surface moist bloody fluid, with few bubbles oozing out on pressure; bloody pleural and pericardial effusions; pluerae, pericardium and peritoneum cloudy and studded with small hemorrhages; spleen enlarged, emphysematous, putrid; liver congested; kidneys with hemorrhagic blebs up to the size of a small beechnut scattered over the surface; intestines much decomposed, so that the presence of ulcerations cannot be affirmed."

Even there he admits the possibility of the so-called ulcerations of the intestines, which is the generally accepted characteristic lesion of hog cholera, and which in my opinion is diagnostic of hog-cholera. In a second animal he admits the presence of ulcerations in the intestines, but at the same time says, in conclusion, "the two epidemics studied by me were due to a small bi-polar germ identical with sample of swine plague received from Washington and culture of Lœffier's germ from Schutz, per Billings. No hog-cholera or swine-plage bacilli occurred. No characteristic lesions have been observed, the pathology pointing to a general infection tending to chiefly affect the lungs and intestines." It seems to me very evident, that these animals were affected beyond all possibility of a doubt with hog-cholera as well as with swine-plague, which the author of this volume claims was the sole cause of death. I do not think he could prove that hog-cholera was not present, unless he examined carefully the several colonies in the Esmarch tubes or plate cultures from each organ, in that way demonstrating the absence of the cholera germ.

I simply say this not as against the work here quoted, because it is such a very good contribution, but as simply showing the difficulty surrounding the investigation of these complicated diseases of swine. It is by no means an easy task as demonstrated by the literature upon the subject.

*The Secretary.* Mr. President, I do not care to switch off from the hog subject, but Dr. Peters this morning in his admirable report touched upon the standards of the various Veterinary schools in the country. I think that the suggestions outlined, that this body, being the leading body should demand for its membership the highest and broadest curricullum and the highest and broadest qualifications of individual members is no longer a question for us to decide. I think the multiplication of our membership as rapidly as it is multiplying is the best evidence of it, and the question is as to whether we are as an association leading the profession in this country. It looks rather that we are willing to follow whatever a school or organization may lead. Certainly we are all imbued with the fact that no one can be learned in the breadth and scope of Veterinary medicine and Veterinary science in less than three years course of six months each devoted entirely to the subject of Veterinary science and no collateral branches. (Applause).

Then it becomes prudent at this moment for this association not through her Comitia Minora, which has seen the failure of members for years, but it becomes better, that this association as a body shall take steps at this meeting to make that a compulsory part of the qualification of the future members of this association, and perhaps no year father away than 1892 for us to adopt it. The multiplication of schools and Veterinary chairs in Agricultural colleges is frought with the greatest dangers to the Veterinary profession of America, and perhaps has retarded us more and kept us in the the position we occupy in the Veterinary world than any other one thing. This association should no longer look to what should be the qualification of a member in a state or local association, but high and above it all should place its own qualifications, regardless or any other organization, on the plane that Dr. Peters has pointed out so clearly. I should like to see a resolution incorporating a suggestion that three years course of not less than six months each shall be devoted entirely to the study of Veterinary science and be one of the necessary qualifications for admission to this association in 1892. (Applause).

*Dr. McLean.* The Comitia Minora were almost a unanimous body for the recommendation for adoption by this association of the very features that have been so carefully laid before it by Dr. Peters in his report. To carry that into effect it would be necessary at the same time to give notice of an alteration of your By-Laws. The By-Laws itself calls for certain requirements and those provisions would have to be annulled in order to put in force the suggestion which has been thrown out by Dr. Peters and Dr. Hoskins. As I said before the Comitia Minora and that was whether it would not be advisable to accept in lieu of one course of six months the certificate of a year's pupilage under a practitioner of our profession. In many schools theory is carried to an extreme point where the practical is very much neglected.

Theory and practice must go hand in hand, and we know that in many cases practical knowledge is only obtained at the expense of the owner of the patient after the pupil has left the school.

*Dr. Michener.* I do not think we are likely to come to any agreement on the subject. There are certainly a few among our Veterinary schools, which, while they claim their willingness to make the course three years, have no idea of doing so. I doubt if they ever will do it, notwithstanding their expressions of willingness, and I think we had better be careful in introducing anything of this kind in our Constitution. I scarcely believe that any of those now operating under a two years course will change it. They say they will do it, but they do not do it. What I think really ought to be done in this matter, and I think our Constitution allows it, is to examine any applicant whether he be a graduate or not before we elect him as a member of this association. Examine him no matter from what college he may come so that we may be sure his requirements meet our ideas of what they should be. I urge upon the Society the necessity of doing this. We could thus get men who would do us some credit. We should insist in all instances on an examination. Some schools with a three year's course turn out men who are less fit to practice the Veterinary profession than some colleges having only a two year's course. Two years of course is to brief a period to study a profession like this. We are or should be studying the profession as long as we live. We only achieve in the short time devoted to the college course the right to possess a diploma. It is after that that we get the real knowledge we possess. I would urge that in future these examinations be insisted upon in a vast majority if not in all cases rather than change the Constitution and By-Laws.

*Dr. Lyford.* We have worked along with little results from the colleges. It is high time that we take another step, and I think that we can take this in advance of the colleges, and they certainly need some prompters. We have asked them various questions and they evade the answers. They tell us that they will do certain things, as Mr. Michener said, and they never come to time. The large proportion of them that have two years course admit that they would be glad to make the course three years. If we can in any way compel or stimulate and induce them to make a three year's course, we have started in advance in a way that will promote the quickest results. It is hardly expected that the professors of the different colleges will get together and agree upon a course, and I think if they see that the standard required by the profession and the U. S. Veterinary Medical Association is a three year's course it will not be long until those now requiring a two year's course, or a practical course of one year and six months or lesss will soon make a three year's course necessaey.

It is not the college, it is the brains that go into the schools. It is the men that make the students, and if we get good students from a two year's course college it is very poor judgment to think that the same good students would not excel in a three year's course college. I do not think Mr. Michener considers that the same student in two years can equal the student for a three year's course, and I think it is a poor argument to advise the continuing of the two year's course if we can in any way remedy it and induce or promote the three year's course principle, and prevail upon the different colleges to come to that standard.

There is another thing. The curriculum of some of these colleges do not require the same amount of studies as others. I think the Comitia Minora will also play a part in this result. When it is brought up, I think we ought to make it strong enough to cover the ground for Agricultural schools and compel them to take an active part as those making a profession of it.

[illegible] *Winchester*. Several years ago there was a By-Law that mem[illegible] should pass an examination in order to become members of this [illegible]sociation, if they did not possess a diploma of some school. That sounds first rate. I had the misfortune to be by proxy at one time an examiner of some gentlemen who were proposed for membership. That examination was a farce. It was over in less time than it takes to tell about it, and the parties were duly accepted as honored members of this body, which undoubtedly they have proved themselves to be by their actions. Now, in order to make an examination necessary for admission to this society and have it straight, and have your examiners chosen from this society, if it is any sort of an examination it will take a week at least, and how many members are there here who will give up one week's practice and come and sit in a hall and keep those fellows from nigging to pass their examination to become members of this association.

*The Secretary*. We had at Chicago some ninety applications for membership, and we did not get through until after two o'clock in the afternoon, and instead of convening at ten in the morning, we did not meet until half-past two in the afternoon. The question of an extended course of instruction of three years' of not less than six months each is to guard against the establishment of a Veterinary chair in which a Veterinary department is in connection with the Agricultural school. They all have their course of three years of six, eight, or nine months each, and at the same time two-thirds of that period is applied to branches that do not directly pertain to veterinary science, and the entire course of instruction, as has been well said by Dr. Peters this morning, is invariably the result of one man's work, perhaps assisted by two or three of his own production. Surely that is not right, and surely as a man coming simply with a diploma of that type should not be considered qualified to be a member of the United States Veterinary Medical Association. (Applause).

*Dr. McLean.* I would like Dr. Michener to understand that in the remarks I make I do not pretend that the United States Veterinary Association is legislating for the colleges, but that they were simply establishing a stand point to be strictly carried out as to the qualifications necessary to be a member of our Association. Let the colleges follow or let them stay out.

*Dr. Clement.* It seems to me that an institution having but two or three professors in a special line may possibly be as good as an institution which turns out men in one very short [illegible]sion; where the principle is that it makes no matter who cor[illegible]n and it is only a question of numbers, and not quality. It s[illegible]ms to me that the one man institution is certainly as preferable as the short course institution. But I am quite in accord with his remarks if a resolution should be passed stipulating the length of a course and the number of professors. If the length of course be stipulated so should be the number of teachers. It seems to me we are going a little beyond our limit. This is a cosmopolitan association, and the question is whether it does not represent the association as it stands, not as we wish it ought to be.

*Dr. Martinet.* Does not this discussion pertain more to the report of Dr. Lyford? It does not pertain to Dr. Peters' paper.

*The President.* Is there any further discussion on Dr. Peters' report ; otherwise Dr. Peters has the floor in response.

*Dr. Peters.* All I will say about my paper is, that I considered everything I had to say very carefully before I said it, and there is nothing in it but what will hold water, and nothing that I will take back. (Laughter and applause).

*The President.* Then we come to the discussion of the paper on diseases. If there is no discussion on that, we will pass to the next paper, which is that coming from the Special College Committee.

*Dr. Martinet.* I think the matter was so ably discussed in paper that we are not able to say much about it.

The Report of Special Committee of Food Inspection was next discussed.

*The Secretary.* Inasmuch as there seems to be an absence of those who have been paying special attention to the subject of that report, that it would be well to receive the report, tender a vote of thanks to the committee, discharge the committee and allow the matter to lay over for discussion in 1892. In the meantime we shall have an opportunity of reading and studying the matter, and then certainly we would be better able to understand the discussion that might take place between those who have made this work a subject of study.

The suggestion of the Secretary was adopted.

*The President.* The next paper is that submitted by Dr. Miller.

*The Secretary.* I will here give notice that to-morrow I shall introduce in writing for the consideration of the association, that it may be brought to a direct vote, a resolution as to whether we shall not raise the qualifications for entrance into this association.

*The President.* We now have before us the report of the Committee on Army Legislation. You heard Dr. Miller's Report. *Dr. McLean.* I move that the report of the Committee on Army Legislation be received and the Committee discharged. *The Secretary.* After all the work that has been done in the matter to discharge that Committee! I think not. *Dr. Miller.* My recommendation was that a new bill should be framed, or amendments offered to the old one, in order that the work be continued, but I hope and pray that somebody else will be put in charge of its continuation. *Dr. Faville.* I move that the report be received and that the Committee continue. *Dr. McLean.* I ask whether it is not a fact that all Committees are re-appointed annually by the incoming President? *Dr. Miller.* I move that the report be received and adopted. *Dr. McLean.* The report has already been received, but I want it adopted because in doing that we adopt the recommendation of Dr. Miller. A new Committee will be appointed and a fresh bill drawn up to meet the objections of the present one. *The President.* The present Committee is in existence until a new Committee is appointed. *Dr. McLean.* My motion is to adopt the report of the Committee and that the Committee be discharged with thanks.

*Dr. Miller.* I might say a word in relation to the work that I was not able to state in the report. As I just stated to Dr. Michener, in the three visits I made to Washington I worked diligently upon the subject. I also corresponded with almost every Senator and Member of Congress whose name and address I could obtain I wish to say also that you have no idea of the amount of labor and time that is necessary to bulldoze--as you might term it—a bill of that kind through Congress in the face of the objections that have been urged against his passage, and some of them very plausible ones. Some of these objections are in letters received from men who have been in the service ten or a dozen years. It does some pretty hard, even if they are not graduates, after they have given their time and services that they should be summarily dropped. If they have been capable of serving the Government all this time, and the Government has received their services, it seems rather a hardship that they should be thrown aside and others put in their places. All they ask is very reasonable. As written in their letters, they asked that they be retired with certain pay. There is one cause of dissatisfaction that we would not have to contend with it it were taken out of the proposed bill. It is hard to put a bill through Congress that is so unfavorable in the eyes of members as this bill is.

As I stated in my report there is a decided objection on the part of Congress to increasing the commissioned officers of the Army.. They say that the Army is too large already, and as these men have done the work heretofore and the Government has gotten along very well with them they do not see the necessity of passing the Bill. That is one of the points brought against it, and when you appear before the Military Committee they have such powerful arguments against the increase of commissioned officers that they make you feel at sea.

I did all in my power to increase the Veterinary force of the Cavalry service. Members of Congress say that they are cutting down the number of medical officers of the army. I think however if the amendments I recommend are made to the old bill that with the united effort of Veterinarians with their Congressmen at home, we can go to the next Congress and get the Bill through without very much legislative work, and it was for that reason I made the recommendations referred to. After having gone all over the ground and given the matter careful thought, I believe from conversations that I have had with members of Congress we could push the matter through.

The President vacated the chair, the Vice-President presiding.

*The President.* I was sorry while in the chair not to see any one take up this question of Army Legislation, because I think it is a very important subject, not only to the community but to ourselves, to this association and to the standard of the Veterinary profession. I have had a good deal of experience, having been Chairman of that Committee for the year preceding the last year, and am thoroughly cognizant with the difficulties which Dr. Miller has mentioned to you, and which I did not mention in my report a year ago as Chairman of that Committee for the reason that at that time the Bill was in such a position I did not care to use names personnally, but which I will do now without hesitation.

It is certainly an outrage that the animals of the United States Army are the only animals in the world that have not authorized official Veterinary protection. Even the Government of Guatemala, a small Republic, has a Veterinary Corps to look after the animals belonging to the Government. There is not a civilized Government, but our own that does not employ a Veterinary Corps. You know the position that a Veterinarian occupies in our Army to day. It was a bone of contention a year or two ago. I could give you the case of a Veterinarian who was sent by the Quartermasters Department of the Army to the West. The letter is on file in the Surgeon General's office to-day, and I have read it. He was in St. Louis five months under pay, reported to the Quartermaster's Department daily, and he did actually nothing. He was sent into the Kentucky to report to a Board for the purchase of horses, where the *Inspector*, who had a great deal of personal ability, looked over and bought a number of horses. The Veterinarian was a thoroughly competent man and former member of this association, now dead. He was not allowed to express an opinion on the animals purchased. They were sent to one of the Cavalry companies and five of the horses the Captain refused to accept, as not fit for the purpose which they were purchased.

The men employed in the army are, some of them, members of this association, and there are some decent reputable men among them that we are glad to have. There are others that you would not employ in your infirmary, and it is among that class that we meet the greatest opposition in the matter of legislation. It is these particular individuals whom officers in the army do not want to receive a commission. I knew all this a year ago, but did not mention it. If those individuals who happen to hear my remarks wish to apply them to themselves, all the correspondence is at the disposal of the association. There are about 150 letters from these men to myself and to each other and letters that have passed two or three hands with endorsements before they reached me, on file and if parties wish to apply to themselves what I am saying they can have the benefit of reading the letters.

Now that is the position of the opposition. It does not apply to these men as a class, but to individuals among them, and it is from that little group that the principal opposition comes. It is going to be slow work for the reasons Dr. Miller has given you. There is a large number of commissioned officers in our army compared with other armies and the disposition of Congress at present is to reduce the number. If we suddenly had a war it would be very easy to get a Veterinary service and have it put upon a good standard. If war was declared suddenly the Government would probably give us fair rank, but we hope of course that there will not be such an event as war. In the meantime we hope to attain our aims and can only work towards them slowly. If it never got beyond the Military Committee every Congress we should have a Bill pending that we think we can have passed and get it endorsed by the head of the army. Certainly a Committee should be kept in this association that will always be acting on the matter or Army legislation and be in a position to take advantage of the first opportunity of establishing a proper service. (Applause.) The President resumed the chair.

The question was put and was received.

*The President.* The next report for discussion is that of the publication Committee.

*Dr. Martinet.* I move the report be accepted with thanks. The motion was agreed to.

*The Secretary.* There is one suggestion I made that some notice should be taken of, and that is the publication of the proceedings of the association. Whether it should be still left with me to make some arrangement with one of the journals for reprints or other arrangements be made. It is a question that onght to be considered under the head of the Publication Committee Report.

*Dr. Winchester.* I move that this association enter into a contract with the publlshers of the *American Veterinary Review* to publish the full proceedings of this meeting. That we make some contract price with them on account of the interest they took last year in our deliberations and the publishing of our proceedings gratis.

*Dr. Williams.* I am opposed to any such arrangement. We cannot afford to show any favoritism between the two American Veterinary journals, and I move that the *Comitia Minora* be instructed to have the proceedings and papers of this association printed and distributed to members. Those taking an active part in these meetings should have a few extra copies. How many copies are needed?

*The Secretary.* We had three hundred and fifty copies from the Review, and I submitted to the Review a list of all our members. These three hundred and fifty copies, with the exception of thirty or forty, have disappeared from my shelves.

*Dr. McLean.* There are only two journals in the United States devoted to our profession, and I do not think we should make any invidious distinction. They will both publish the proceedings in full and we should divide our patronage by getting three hundred copies from one and three hundred copies from the other.

*Dr. Martinet.* I think the best way would be to purchase a certain number of copies from each of these publications, as suggested by Dr. McLean.

*Dr. Miller.* I agree with Dr. McLean that we should make no invidious distinctions. I think the editorial staff of one journal is just as much interested in us as the staff of the other journal, and one has done as much for the advancement of our interests the other. I therefore second the motions made by Dr. McLean.

*The President.* If you will allow me a suggestion, I will say that if the association wishes to assume the expense of publication, why not do it on business principles, and ascertain where they could get the number of copies they desire at the cheapest rate.

*The Secretary.* The expense of the Stenographer's Bill at Chicago was divided, and my trouble was to furnish to each one of them the manuscript so that they could get their journal out at the same time with the same report. One paper was setting up the type while the other did not have the copy. I felt as much interest in one journal as in the other, and the thing became a source of trouble. We always allow fifteen or sixteen days to get the matter up in type, which is a short period.

*Dr. McLean.* I renew my motion that this association purchase three hundred copies each from the Journal and the Review of the entire proceedings of this association as printed in those publications.

*Dr. Winchester.* Where are you going to buy them? *Dr. McLean.* From the publishers.

*Dr. Clement.* Are we to give these away to those who should subscribe to the journal, but who do not do so? *The Secretary.* I have always taken advantage of the opportunity to send to the young men all our reports gratis.

*Dr. Berns.* Last year was the first time that our reports were formulated in anything like proper shape. Heretofore we simply had a synopsis of our proceedings. The entire proceedings from beginning to end were duly recorded at the last meeting. It seems to me that simply a synopsis, which is all we could expect from either of the journals, is hardly sufficient for our purposes. As long as we have a stenographer here a full report should be printed and paid for by the association independent of the journals. That would be the correct thing.

*Dr. Williams.* The proper thing is to have an entirely independent report of the proceedings of this association that every member of this association may have a copy, and then the editors of these journals can make extracts and such comments as they see fit. It seems to me that is in every way the better plan. I, however, take the *Review* and the JOURNAL, and would not be without them. I prefer to have the comments of the *Review* and JOURNAL, and then have a special copy that I could put upon my shelf as the report of this meeting of 1891.

*Dr. Clement.* I move as an amendment to the motion of Dr. McLean that the publication committee of this association be authorized to have printed five hundred copies of the proceedings of this association for distribution among the members without regard to either the JOURNAL or *Review.* In other words to have the printing done where it can be had cheapest. *Dr. Peters.* I would suggest as a substitute that the association allow the journal offering the lowest bid the privilege of publishing the proceedings of the association. *Dr. Miller.* If Dr. Clement will offer his amendment as a substitute I will accept and vote for it. *Dr. McLean.* Can you give us any idea of what the cost will be. *The President.* If you base it upon the proceedings of last year, I

assume that the cost of printing the proceedings will amount to a little more. If the association had undertaken to publish the proceedings itself last year the cost would have been about $170 after they got the copy. I mean that is the cost not including the first expense.

*Dr. Clement.* Cannot the publication committee have this printed by one of the journals. The journals are not disbarred. I simply mean for the publication committee to have it done wherever they could have the work done cheapest without regard to any particular journal. *The President.* Exactly, the cheaper it is done, the better for the association.

*Dr. Miller.* And then it does not appear on the minutes that we draw an invidious difference between one journal and then the other, let the committee get the work done where they can get it done cheapest. The question was put on Dr. Clement's motion and it was agreed to.

*The President.* You have now before you the matter of the Special Committee on Central Organized Body, which made no report.

*Dr. Winchester.* That committee has been in existence, if I remember rightly, four years. There never has been a report from it. Is has never been demonstrated to this society what the standing of that committee was. I, therefore, move that the committee be discharged and not another one appointed. *The President.* It is a special committee, and if discharged it would require special action in order to appoint another. The question was put and the motion of Dr. Winchester was agreed to.

*The President.* Is there any discussion on the report of the Special Committee on food inspection? *The Secretary.* That goes over to the next meeting for discussion I believe. *The President.* You now have before you the Secretary's report. *Dr. Winchester.* I move that it be accepted, except as to the last clause. (Laughter and applause). The motion was agreed to.

*The President.* Have you any action to take on the reports of the Assistant Secretaries? *Dr. Miller.* I move that they all be accepted and filled. *Dr. McLean.* Coupled with a vote of thanks. The motion as amended was agreed to.

*The President.* The next order of business is the election of the officers, which concludes the business for the day. To-morrow being set aside for the reading of papers. *Dr. Winchester.* I nominate for President, Dr. J. H. Stickney, of Boston. *Dr. McLean.* I second the nomination. *Dr. Miller.* I nominate Dr. R. S. Huidekoper for re-election. *Dr. Kuehne.* I second the nomination. *Dr. Clement.* I nominate Dr. Williams. *Dr. Berns.* I second the nomination. *Dr. Kilborne.* I nominate Dr. Michener. *Dr. Lowe.* I second the nomination. *The President.* I have decided not to be a candidate, and you have before you the three names of Drs. Stickney, Williams and Michener.

*Dr. Michener.* To make matters a little easier I withdraw my name, thanking you gentlemen for the honor. I could not possibly attend the duties of the office. I respectfully decline the honor.

*Dr. Miller.* Before we go into the election of officers, I want to rise to a question of privilege. I would like to know whether or not in the absence of a member of this association, such absent member can be nominated for the office of president and elected?

*The President.* There is certainly no regulation against it.

*Dr. Miller.* It seems to me to be a very unwise thing to do. I have nothing against Dr. Stickney, and if here, I would vote for him, but I think if a man has not enough interest in this association to attend its meetings he ought not to be put up by his friends for president of the association. I have the highest regard for him as a man, and would vote for him if he were here. (Applause.)

*Dr. Winchester.* I nominated Dr. Stickney knowing that he was not here, but if the gentleman will look over the records I think he will find that since the birth of this association he has attended the meetings as regularly as any member of the association. Because he is not here to-day, I do not think it disqualifies him. As a member of the association he has done more than any one else to sustain the work of the profession. He has taken the position that our friend Hoskins always has taken as regards the education of a man to be a veterinary surgeon, and he has been to us down in Massachusetts a pretty good guide.

*Dr. Miller.* I grant all that has been stated by Dr. Winchester. No man in the profession has a higher regard for Dr. Stickney than I. My question was simply asked as to the precedent. I do not think it is the province of this association to elect a man to the office of president of the association who is not present at the meeting. I did it upon what I considered constitutional grounds. There is not a man in the profesion to-day, not even Dr. Winchester himself who has more respect for Dr. Stickney than I have, but as he is not here I cannot vote for him. I do not think we should elect him in his absence.

*The Secretary.* There is no member of the association that it would give me more pleasure to vote for than Dr. Stickney, but Dr. Stickney for the last few years, because of infirmity of some kind, has not been able to mingle much with us, and we are now approaching an international meeting in 1893, and it is of the utmost importance at this time to elect as president a man that will lead this association up to all the necessary qualifications for that meeting. I think it requires one who has been very closely idendified with all the work that has been specially done during the last four or five years, and who has not only mingled with us, but who has been active on committee work and been associated with the Comitia Minora, therefore I trust that our present presiding officer will not withdraw his name under the circumstances. (Applause.)

*The President.* Gentlemen, nominations are still in order.

*Dr. Winchester.* Under those circumstances, I most respectfully withdraw the name of Dr. J. H. Stickney.

*Dr. McLean.* I second with deep regret, feeling that the association has expressed the sentiment concurred in by the mover of his nomination, and I withdraw my second.

*Dr. Berns.* Then the only candidate for the time being, as I understand, is Dr. Williams. *Dr. Winchester.* Dr. Huidekoper and Dr. Williams. *Dr. McLean.* I move that the nominations be closed. The motion was agreed to. *The President.* I will appoint Dr. Winchester and Dr. Rayner as tellers. The tellers took their places and the result of the ballot was announced as follows: Dr Huidekoper, twenty votes; Dr. Williams, six votes; Dr. Michener, two votes. The election of Dr. Huidekoper was made unanimous.

*The President.* Gentlemen, I am very thankful to you for this honor. I meant what I said when I declined to be a candidate for the office of president. The special reasongiven is the only reason that I allowed my name to be used, namely: that during this year the work to be done will be largely preparatory for the Chicago meeting, which we have decided to make international in character, and I know probably more veterinarians personally than many members of the association, I am willing to aid in the work.

Nominations for Vice-President are now in order.

*Dr. Winchester.* I nominate Dr. W. L. Williams. *Dr. McLean.* I second the nomination. *Dr. Martinet.* I move that the Secretary cast the ballot of the association for Vice-President and Secretary and Treasurer in favor of the incumbents. The motion was agreed to. *Dr. McLean.* Now I move that the Secretary cast the ballot of the association in favor of Dr. Williams for Vice-President. The motion was agreed to, and the Secretary cast the ballot as directed, and announced that Dr. W. L. Williams has been elected Vice-President. On motion, the Vice-President cast the ballot of the association for Dr. W. H. Hoskins as Secretary. *The Secretary.* I again ask that I be relieved of the position. (Cries of "No, No!")

*Dr. Martinet.* I move that the Secretary cast the ballot of the association for Dr. Robertson for Treasurer. The motion was agreed to, and the Secretary cast the ballot as directed, and announced that Dr. J. L. Robertson had been elected Treasurer.

*Dr. Winchester.* Under the head of new business, I wish to revert to the subject that was discussed to-day with regard to refunding to our friend, Dr. Hoskins, the discrepancy of last year's salary. I move that he be refunded fifty dollars.

*The Secretary.* I object to that. It certainly will not appear on the minutes that my asking you to enlarge the salary of the Secretary was a sincere thing, especially when you follow that up by making a donation which he has not asked. I still insist that I am not willing to serve again as Secretary, and think I have a right to be heard. I have served you three years, and that is as much as you ought to demand on of any one man. *Dr. Faust.* I I think the demand is just and right, but I also think that to accede to the demand would be fatal to the society. *Dr. Miller.* I hope that the remarks of Dr. Hoskins will not be taken in the sense in which he has intimated. It is not that way. The President and myself know that the amount he received last year did not begin to pay his expenses in any way or shape. I know the hours that he has spent in correspondence. It has been almost impossible for me to get an audience with him at home for the reason that he was so busy with the duties of his office. It is not that we want to give him back pay, but simply to reimburse him for expenditures that he has made on our account, and I hope the motion will prevail. The question was put, and the motion made by Dr. Winchester was agreed to.

*Dr. Miller.* I move that we do now adjourn to meet again to-morrow at ten o'clock. *Dr. McLean.* I second the motion.

The motion was agreed to, and accordingly, (at five o'clock and forty-five minutes p.m.), the meeting was adjourned.

*Secretary's Statement for Year ending September 14th,* 1891.

| | |
|---|---|
| To annual appropriation for banquet and deficit | $ 78 00 |
| To rental Auditorium Hall | 60 00 |
| To L. McLean, expenses, Com. on Tuberculosis | 18 00 |
| To C. R. McKenzie, Dist. Pass. Agent, B. & O. | 28 70 |
| To Secretary, salary, one year | 100 00 |
| To printing | 32 25 |
| To postage, stationery, expressage, etc., etc. | 74 51 |
| Total expenses for year | $391 46 |
| By collection of initiation fees and dues, Banquet fees, Two-third expense of stenographers, Charges repaid by *Review* and JOURNAL | $654 50 |
| Balance in Secretary's hands | $263 04 |

Signed, R. A. McLEAN,
THOS. B. RAYNER,
AUSTIN PETERS,
*Finance Committee.*

---

SEPTEMBER 16TH, 1891; 10 O'CLOCK, A. M.

Convention met pursuant to adjournment.

The Secretary called the roll with the following result: Members present: Drs. Barron, Berns, Bryden, A. H. Baker, S. S. Baker, Claris, Clement, Dougherty, Wm. Faust, Faville, Hinkley, Hitchcock, Hoskins, Huidekoper, Kuehne, Kilborne, Kidd, Lyford, Lowe, Martinet, Michener, Miller, R. A. McLean, F. W. McLellan, Peters, T. B. Rayner, Jas. B. Rayner, Jas. L. Robertson, A. K. Robertson, Swedburg, Thompson, Turner, Wende, Weber, Winchester, Waugh, W. L. Williams, Meisner, Knowles.

As delegates: Drs. J. C. Dustan, New Jersey; R. G. Webster, Pennsylvannia.

As visitors: Drs. Isaiah Michener, Jno. W. Gadsden, N. Rectenwald, H. S. Hogsett, H. B. Rayner, A. M. Farmington, W. H.

Scrubey, W. Runge, Wm. Somerville, F. E. Parsons, C. B. Robinson, J. D. Robinson, Wm. B. Werntz, G. A. Jaman, I. N. Krowl.

*The President.* There is still some unfinished business to be transacted.

*The Secretary.* I have received a letter from Dr. Whitney with a check for his back dues, and I move that we rescind the action of the association yesterday in adopting the recommendation that he be dropped from the rolls, and further move that he be reinstated as a member of this association.

The motion was agreed to.

*The President.* The Secretary has another matter to submit.

*The Secretary.* I have to offer the following applications properly authenticated for honorary membership :

### *Applications for Membership*, 1892.

HONORARY MEMBERSHIP: William H. Welch, M.D., Professor of Pathology, Johns-Hopkins University, engaged for the past four years in the investigation of infectious diseases of animals, especially of swine, endorsed by the members of the Maryland State Veterinary Society. Proposed by Dr. A. W. Clement and seconded by Dr. Rush S. Huidekoper.

FOR REGULAR MEMBERSHIP :

Dr. I. N. Krowl, (Am. Vet. Coll.) Passaic, N. J.; Vouchers, F. F. Winchester, Austin Peters.

Dr. William B. Werntz, (Vet. Dept., U. of Pa.), Philadelphia, Pa.; Vouchers, R. S. Huidekoper, S. E. Weber.

Dr. A. M. Farrington, (Cornell Vet. Dept.), Washington, D. C.; Vouchers, Chas. B. Michener, William F. Howe.

Dr. Fred W. Ashe, (Chicago), Chicago, Ill.; Vouchers, A. H. Baker, S. S. Baker.

Dr. Theobold Smith, (Albany Med. Coll.), Washington, D. C.; Vouchers, Chas. B. Michener, W. L. Williams.

Dr. F. K. Choffee, (Chicago), Chicago, Ill.; Vouchers, A. H. Baker, S. S. Baker.

Dr. H. W. Hawley, (Chicago), Chicago, Ill.; Vouchers, A. H. Baker, S. S. Baker.

Dr. G. Allen Jaman, (Am. Vet. Coll.), Chestertown, Md.; Vouchers, A. J Thompson, J. A. Kuehne.

*The President.* I believe that the Secretary has received a report from Dr. Butler.

*The Secretary.* I received from Dr. Butler the report of the Committee on Diseases.*

Gentlemen you have heard the report of Dr. Butler. If there be no objection the report will be received for discusssion later on.

*The Secretary.* I have the great pleasure of announcing the application of Dr. Theobald Smith for membership.

*Dr. Kilborne.* If it is in order I volunteer the information that if any members of the profession would like to see the Texas Fever Entozoon they can do so by calling at the laboratory of Dr. Smith who would gladly show it to them.

*Dr. McLean.* In accordance with the notification given yesterday, I desire to offer now for consideration at the next meeting the following amendment of the By-Laws :

Article 1 reads that "any applicant for membership shall have his name proposed in writing by a member of the Association in good standing who shall furnish evidence of the fact that he is, first a graduate of a regulary organized and recognized Veterinary or Medical school ; second, that he is of moral and reputable business methods."

The proposed amendment is to strike out that article except the last clause and substitute this :

"Article 1. Any applicant for membership shall submit his name upon one of the association's application blanks, duly vouched for by one or more members of the association, or by the resident State Secretary of his respective State. He shall be a graduate of a regularly organized and recognized Veterinary School, which shall have a curriculum of at least three years, of six months each, specially devoted to the study of Veterinary science, and whose corps of instructors shall contain at least four Veterinarians. If of a medical school a similar curriculum as to time shall prevail."

This alteration to go into effect after the annual meeting of 1892 it shall not be retroactive nor apply to applicants who were college matriculants prior to its passage.

*The President.* I will ask Dr. Kilborne to repeat his invitation, as there seemed to be considerable interruption at the time I do not think that everyone heard it.

*Dr. Kilborne.* I simply announced that if any member should like to see the Texas Fever Entozoon, and they will call at the

* Will appear later.

Laboratory of the Bureau of Animal Industry of the Agricultural Department I know that Dr. Smith will gladly show it to him. I would also say that if any of the members of the profession would like to see the blood of Texas Fever animals that we have several chronic case at the station here at present, which are open for their inspection.

*The President.* We will now hear the report of the Finance Committee.

Dr. Rayner read the report as follows, which has received and approved :

JAMES L. ROBERTSON, Treasurer,

In account with the United States Veterinary Association.

| | | |
|---|---|---|
| Balance on hand, September 16th, 1890 | | $703 43 |
| Paid Bennett, Edwards and Pettit, stenographers | $ 81 50 | |
| October, 10, 1890, paid Chairman R. S. Huidekoper, Committee on Legislation | 166 50 | |
| | | 248 00 |
| | | $455 43 |
| In bank | $447 18 | |
| In Treasurer's hands | 9 25 | |
| | $455 43 | |
| Received from Secretary | 36 81 | |
| Balance due per audited account | $492 24 | |
| In hands of Secretary, Sept 1st, 1891 | 263 04 | |
| | | $755 28 |

Approved, R. A. McLEAN,
THOS. B. RAYNER,
AUSTIN PETERS,
*Finance Committee.*

*The Secretary.* I have the following charge preferred by Dr. Faust :

I herewith prefer charge against Dr. John T. Claris, of Buffalo, N. Y., for manufacturing proprietary and brevet medicines and unprofessional methods of advertising as per proofs at hand (proofs filed) and would recommend that his certificate be with held. (Signed) JOHN FAUST, Poughkeepsie, N. Y.

*The President.* The charge will be referred to the Comitia Minora and go through the regular course. If there be no further new business we will proceed with the reading of papers. The first is by Dr. Lyford on "Barren Mares." *

* Will appear November number of the JOURNAL.

After the reading of the paper, the discussion was deferred in order to allow Dr. Bryden to read his paper on the Transatlantic Cattle Trade, and its regulations from a Veterinary point of view.

See page 495.

*Dr. Michener.* I have the pleasure of extending to you a hearthy and cordial invitation from Secretary of Agriculture Rusk to make a visit to the Department. You will be able to see much to interest you in many ways. The invitation embraces the experiment station under charge of Dr. Kilborne, a member of this Society. You will be able to see the clinical work he has done. Everything connected with Department we would be glad to have you investigate. The Secretary told me to be very particular to ask each and every one of you personally to come, and I take this means of doing so.

*Dr. Faust.* At what hour? *Dr, Michener.* Any time before four o'clock. At this point (thirty minutes past twelve o'clock P. M.), the convention took a recess until half past one o'clock P. M.

AFTER RECESS.

At the expiration of the recess the convention resumed its session.

*The President.* The next order is the paper of Dr. Williams on "Rachitis." See page 477.

*The Vice-President.* We will now hear the paper of President Huidekoper on the "Identification of animals." *

*Dr. Lowe.* Before we proceed with the discussion of papers I would like to refer, if it is in order, to an invitation that we have received to-day, as there should be some action taken on it, and that is the invitation of Dr. Michener. (Cries of "Too late to-day!") It may be too late to-day, but we have received a very kind invitation from a high authority through Dr. Michener, to visit the buildings of the Agricultural Department, and I think it would be fitting and proper to accept Secretary Rusk's invitation and visit the buildings referred to in a body, and I would make a motion to that effect.

*The Vice-President.* The motion would scarcely be in order at this time. It is too late now.

*Dr. Lowe.* I refer to to-morrow morning. I make the

* Will appear November number of the JOURNAL.

motion that this association accept the invitation of Secretary Rusk as made through Dr. Michener, and we visit the Department to-morrow morning at ten o'clock as a body. The motion agreed to.

The Secretary announced the application for membership of G. Allen Jaman of Chestertown, Maryland.

*The Vice-President.* We will proceed with the discussion of the reports and papers. The first in order is the discussion of Dr. Butler's report from the Committee on Diseases.

*Dr. Faust.* One of his assertions was that Dr. Koch has claimed to cure tuberculosis, which statement is wrong and must be stricken out of Dr. Butler's paper. He has never made that statement, never made such an assertion either publicly or privately, as I have read. *Dr. Waugh.* I second Dr. Faust's motion to strike out that remark in the paper of Dr. Butler. *The Secretary.* Dr. Butler simply made a criticism of it and made no absolute statement. *The Vice-President.* Dr. Faust's statement will simply go on record as denying a portion of the report. *Dr. Lowe.* I do not think we have any right to change Dr. Butler's paper. We may of course comment on it.

*The Vice-President.* We have not any right to take any statement out of the report of Dr. Butler. If there is no further discussion on that paper the next paper in order is that read by Dr. Lyford.

*Dr. Faust.* Before I came to this place, I saw that there was a paper to read on the subject of "Barren Mares," and naturally I looked up the subject of "Barren Mares," and naturally I looked up the subject to get something that might possibly be of value in this gathering. I found that a man named Friedler was asked by the German authorities why he was so successful, and so much more successful than the rest of the breeders, and he claimed that his success was by reason of the appropriateness of the time when copulation took place. He says that he never had the mare covered—that is the particular mare I am speaking of—excepting on the third day after the heat began and then on to the seventh. From the seventh day, if the mare has not conceived, to the twenty-first and never to return again until the next period. He separates his mare from his horse and recommends very highly not to give too laborious work as to speed or labor and not to feel too high, or as low down as starvation, but to keep the animal in good sanitary eondition, and by no means does he allow his mares to come in contact with a stallion during the time I speak of. To show the correctness of his idea he speaks of seven mares that had a very bad reputation as foal givers. He took twelve mares that had likewise a bad reputation of bearing, one of them over thirty years old, and he reports as a test of his theory that he had eleven foals out of the twelve mares, and he, as I said before, claims that the time or period is worth more than any other theory that has been advanced—of course, diseased condition excluded.

*The President.* I should like to have seen this brought out a little more clearly in Dr. Lyford's paper. I am very sorry he did not give us more statistics as to the results of using the impregnator as a mechanical means of overcoming the troubles of sterility due to mechanical causes. He made a quotation from Dr. T. G. Thomas that applies to the mare quite as thoroughly as to the woman, and that was that the trouble was first in the neck of the uterus and secondly due to the endometritis. That is the discharge of endometritis in place of being alkaline is acid. Spermatozoa cannot penetrate where there is a discharge of acid fluid. It must be alkaline fluid. I think in a majority of cases that that is the trouble. Again there is no question but what mechanical means for the dilitation of the neck of the uterus will sometimes relieve the inflammation, causing a very slight attack of endometritis and act as a curative agent. Just as the passage of a sound into the urethra where there is stricture will do in cases of gonorrhoea. Take gleet, and a sound changes the discharge from that of a chronic inflammation to that of an acute one which frequently subsides at once. I would ask Dr. Lyford if he can give us the statistics as to the use of these dilators.

As it is advertised as a common article and put forth to the public, it is very misleading. I had a case of a very valuable mare that had been under treatment for a year with dilatations, my first examination and syphoning brought out five gallons of fluid. He spoke of using the pumping syringe. In place of a syringe which I rarely use now in those cases, I have an India rubber tube four feet long, in the end of which I have a lead nozzle over which rubber of the same diameter is stretched. With this I can throw in the quantity of water I wish and get the pressure I want. Where there is a large discharge I allow it to go twelve inches into the uterus and pour the water in. In this way I have a syphon which will clean the womb out entirely and throw in the amount of fluid that the walls can stand. I think it a safer instrument than the syringe that he spoke of. I think Dr. Lyford should indicate what percentage of sterility he thinks is due to mechanical causes.

*Dr. Knowles.* I have devoted some attention to sterility for the last four or five years and I believe that a small percentage is due to a constricted os. The most frequent cases of sterility I believe to be following chronic cervical hyperamiæ. In regard to the syringe I will say to Dr. Huidekoper that I have a sort of "universal family syringe" that I use for mares in the shape of a ten gallon keg. Attached to that I have a long piece of soft rubber hose. I can elevate this keg to any distance I desire by a pulley and rope, and give such force to the water as I desire, and as little force as I desire. I simply use to rubber tube without any metal appliance at the end of it. The acute cervical hyperamia I have found to be best treated by injections of cold water at a temperature of 65° F. with from ten to twenty per cent. of boracic acid. Chronic cervical hyperamia is not always amenable to treatment, but in a majority of cases it is.

This is a subject though too long for me to talk about here, and as I have quite considerable data and notes on cases treated, I will try at some future date to give you a paper on sterility according to what I know about it.

*The Secretary.* I was exceedingly interested in the paper of Dr. Lyford on the question of sterility, and assure him that he has enlightened me on many causes that operate in that one disease, that is such a great loss to the breeders of the country. At the same time he has brought in here for us to examine and consider a number of articles which are certainly decidedly out of place. As a body or veterinarians we can give no cognizance to patent articles by the laws under which we operate. That is equally true of proprietary articles, and the remedies here exhibited for us to consider and discuss are certainly out of place. This association cannot be in any way used for the advertising of specific plans of treatment, or of patent instruments, or of proprietary articles. (Applause.)

*Dr. Lyford.* I do not think there is one of those articles patented. At least not to my knowledge. In the second place Mr. Barnes, of Connecticut, took the pains to send me some homeopathic medicines, which if you all know about as I do you will not hurt yourself with. I brought them here thinking somebody had experimented with them, and if they obtained results, I should like to know what they were. I have never tried them. I brought them here because he was kind enough to send them for me to look into if I wished to use them. I know nothing about what they are and know nothing further than their labels indicate. I have a number of testimonials from those to whom I have sent my articles wherein they claim that the articles have given good results. So far as recommending them to you I do not think I have flattered myself or the instruments very much in my paper. The question of simply bringing them before the public is simply to show the principle. If they are wrong in principle, I would like some one to get up and say where and how. Or they can take them out in the dark and feel them. (Laughter.)

*The Secretary.* I referred to a portion of the paper where Dr. Lyford, in speaking of the impregnation, states that it is possible for it to take place without the rupture of the hymen. Does he mean complete or partial.

There were three cases reported of large calculi, or stone, found within the uterus, and one was in an animal that had been served by a horse unsuccessfully. In the other two there was a complete hymen, and the conclusion was that it could not have been put in from the exterior, if such was the case. The stones were not exhibited, but were ordered to be sent to Philadelphia for examination. They were described as weighing somewhere from twelve ounces up to two pounds. There is nothing in the books about it, and I simply ask as to the rupture of the hymen, whether it was complete or partial.

*Dr. Lyford.* I have examined a good many mares, and as a rule, make an examination before the stallion is allowed to cover them. In a great many cases they claimed to have bred the mare prior to this time, and that the hymen was still intact, and in some cases probably not complete. In a great many of these cases as you pass your hand in and bring it back, you can feel the membrane very plainly.

Sometimes you can draw it outside. I have done this and shown it to parties and students who were standing by. I have known a stallion to cover a mare and the upper part of the hymen to remain intact, and the copulation seemed to be complete and all right. As far as a case that had been in foal with the hymen intact, I do not know of such a case.

*Dr. Williams.* I would like to make a few remarks. I have been engaged in practice largely in horse-breeding districts, and mostly with heavy draft animals. In my experience sterility has been due usually to, first excessive fatness and, second, to a plastic condition of the uterus with widely dilating os.

*Dr. Bryden.* I have listened with interest to this matter of the peculiar characteristics of breeding mares. I think we mistake very much when we commence so far up as going to the matter of copulation. The influences of domestication have much to do in forcing changes in the genital organs, and in other parts of the system, and my impression is that instead of devoting too much time, which of course is laudable in trying to correct these defects, that it would be better to improve our system of managing horses under restraint. Of course where animals are now kept in a stable for six months of a year they do not attain to that robust and symmetrical organization that would be attained if they were running around with greater freedom of action. An important fact is to think of the actual condition of things. Animals with defects of this kind are always animals that are not fit to breed from. Much is to be accounted for by the animal's condition.

*Dr. Lyford.* Dr. Williams has brought out a point that I had overlooked in my paper, although I had it down as a point. One point he made was that this band attaches to the os and diverges in different directions. I want to call on Professor Baker for an explanation, so that we may hear both sides of the question.

*Dr. Baker.* In reference to the use of the impregnator, I have no experience, but from my observation I have opportunities of knowing that in many cases it has proved successful and very satisfactory, and I wish to say in regard to an expression of opinion dropped a few minutes ago by the Secretary, that this association should denounce the patenting of instruments, that in my opinion it is a little too finely drawn, and this association as veterinarians, can hardly afford to be too strait-laced in regard to such things. Our interests and the interests of the agriculturalists are closely allied, and anything that is of practical use, whether patented or not, if it is, as I say, of practical use in the advancement of stock raising, we should recognize it and not refuse to give support to an impregnator, for instance, simply because it is patented. It seems to me it matters not whether it is patented or not, if it is useful it should be endorsed. While, of course, this cannot give a positive cure to barrenness in every case, yet we do know that in many cases it has proved very beneficial.

Now in regard to the idea that domestication is responsible for barrenness in some cases. It is possible that in some cases it may be, but we find barrenness in many cases where the animal has not been in anything but natural state. Therefore, in such case, the domestication of course could have nothing to do with the barrenness of the mare. In many cases I am confident that the impregnator may be made very useful.

*Dr. Miller.* I had in mind to reply to our Secretary some moments ago, but I thought perhaps in doing so I might be called to order by the President or some member of the association as being a little out of order, my remarks being foreign to the subject. I could not very well speak as to the impregnator because it is the first time I ever saw one. I think as Dr. Baker does, that our Secretary draws the lines a little too fine. I can see no comparison between the impregnators upon my left and patent medicines in my front I do not see why any member of this association should be taken to task, even if he recommended an article of that kind, or of any other kind, provided it was beneficial to us in our practical purposes and beneficial to us in our profession. (Applause.) I believe that we are as a class of people doing what we consider best for the elevation and standard of veterinary science, and I believe we are not doing anything to lower it when we recommend anything that we know is practical or otherwise of benefit to us as veterinary scientists. It seems from the statements made here that these instruments have been of great benefit. The fact of these things being advertised in the journal at Chicago, or advertised by the proceedings of the society seems to me to be a benefit to the veterinary science rather than the opposite. We are to-day the only people in existence that I know of who do not advertise their business. If any veterinary college, or this association, put an advertisement of the business in a newspaper, excepting the simple fact that the doctor is a veterinary surgeon, and will be found at a certain house, he would be brought before us for court-martial. I say this is drawing the line too fine, and is injurious to our profesion, I believe at the same time that it would be well for us to make no invidious comparison between patents. I take it that upon the whole we should not confine ourselves so considerately to the letter of the law in respect to these matter as they touch the code of ethics.

*The Secretary.* It has been an established rule in this Association by usage, and in the Constitution and By-Laws and code of ethics of this Association from its formation, in 1863, that we take no cognizance of any patented article, or any article in which a member of this Association, or member of this profession, sought a patent for. Any instrument or implement of use that any member of this Association had any knowledge of, or any plan of treatment, specific in character, or of extreme value to the profession, that he may have discovered, it is his duty to impart it to us, and it is our right to demand it. From the usages and laws of this Association and from its earliest growth this has been a cardinal principal with us. We are banded together as a body of men. For what? Not for our individual advancement, nor our individual success, but for the welfare of our country. According to your rules and by-laws the article is patented, we must by virtue of the respect we have for those laws, denounce the use of it. Therefore, it ill-becomes a member of this Association to seek a patent on an article by which he will have sole control of that article. Again, we are amenable to him for its use as well as the man who puts up the proprietary remedy and refuses to divulge its formula and puts it upon the market. The usage of this Association is such that no man is allowed to advertise anything but his name and address, and if he is competent in his profession and is fitted for the duty that he has assumed, and exposes himself to the public, there is no town, there is no city, there is no borough in these United States where he will not receive a just measure of reward for the ability that he possesses, and receive in return a fitting compensation for all that he does towards benefiting humanity, and all the brutes of creation.

*Dr. Miller.* No one appreciates what the Secretary has said better than I do, but when it comes to the poor fellow he speaks of who is cast out in the borough and puts his sign on the window and sits for patients, I know that there will a long time elapse between drinks, and it will be a long way between meals. It will be some time before the people will come to him. He has gradually got to drift into practice and wait for people to come to him. We are the only profession disqualified from advertising, and while I agree that it is right and proper that it should be so, and while if we are deserving of patronage the people will gradually come to us, at the same time I do say that we are drawing the line a little too fine when, as I have heard it expressed here, a man should not even put a thing on his letter-head but his own name. I do not believe in advertising at all, but at the same time I do not believe that because a man puts on his letter-head that he belongs to a State Society, or to this Society, that he is amenable to the strict laws of the Association. I am not in favor of promiscuous advertising, and do not wish to be so understood.

*The President.* The paper on the transportation of foreign cattle and its regulation is open for discussion.

*Dr. Michener.* I wish to say a few words in reply to what the gentleman has said in regard to the regulations that should govern the shipment of cattle abroad. I did not read all of the paper or hear its full reading, but it strikes me that the burden of his song was that while certain restrictions were placed upon steamship companies that similar heathful restrictions were not placed upon the railroad cattle handlers, and should be so placed as soon as possible. I have no reason to question the fact referred to by Dr. Bryden, but take it that the irregularities complained of are only around Boston. We have no such condition in the stock-yards of New York and Baltimore, Philadelphia and Newport News. We have nothing in these yards which appear to be anything like as bad as he details as existing in Boston. I have seen most of these yards I speak of, and instead of the cattle wading in manure up to their knees or bellies, I found the yards to be well paved, to be covered, and cattle protected from the sun. They have plenty of room, too. Another point he makes, is that cattle do not have sufficient rest. I would say that export cattle have, as a rule, twenty-four hours rest at the point of shipment; at least they should have that. If the parties conformed to the rules and regulations of the Department of Agriculture, Bureau of Animal Industry, they would have that rest. That rule is waived in some instances, when they come in a few hours behind time, and where the steamer must sail by a certain hour. Then they go a little short of twenty-four hours rest; but whether they go much short of that I do not know.

In regard to regulations of shipment, as applied to steamship companies, I will say that these rules were only issued after a friendly conference between Secretary Rusk and the Agents of the Transportation lines. The regulations were the result of this conference. There was no objection to any regulation by the steamship people at that time, and I do not see any reason for objecting to any regulation. They seem to me to be eminently fair and proper. Whether those regulations were issued as a humanitarian policy, or a policy of gain, I don't think it is worth while to go into. These regulations were hurried by the agitation on the other side. We all know of the exaggerated statemen made abroad of the cruelty in the shipment of cattle brought over. Upon investigation those charges were found to be unfounded. The export cattle trade to-day is as well conducted, so far as shipments are concerned, as any other trade, and cattle are going over at a less loss at this time than ever before.

Complaint is also made by Dr. Bryden as to shipments across the country, that we do not disinfect the cars fully. I understood him to make that assertion. In reply I will say that we do disinfect all cars.

During the summer, when we were having danger from the Texas or Southern fever, I am glad to say that among export cattle the loss was thirty-three and a third less than heretofor. I hope that in a year or so, by watching all the railroads and stock-yards, we will have nearly wiped it out.

There are very marked improvements that may be made in the transportation of cattle from the interior to the sea-board, and the Department is struggling all it can to bring forward that condition of affairs which we all wish for.

The gentleman spoke of palace stock cars, as they are called. I think that you can understand that the Department of Agriculture cannot champion any one of these cattle car companies and push it on the market, but we are insisting as nearly as we can upon the frequent unloading of cattle. I will state to the Doctor that if he knows of any instance where the cattle are not unloaded and rested and watered at the proper points within the proper hours, he should call the attention of the authorities to it. There is a statute that compels the railroad companies to unload cattle every twelve hours, or something like that. I do not remember the letter of the law exactly, but it is safe to say that the law provides they shall be unloaded every twenty-four hours, or one in every twenty-four hours, and fed, watered and rested. And any body who says the railroad people are violating the statute has nothing in the world to do but bring it to the notice of the United States District Attorney, whose business it is to prosecute the railroad company for such violation. The fine upon conviction is five hundred dollars, and half of the fine goes to the informer. So that if the Doctor knows of these violations he will have no trouble in making some money.

I do not know that I have anything more to say about this matter than I have stated, except to add that cattle are certainly shipped very thoroughly nowadays. I have seen cattle come in

New York by the hundreds of car loads, and I have never seen them come in suffering. The restrictions on the railroads will be more severe in the future, and I have no doubt everything will be done that is practical to do in this great traffic. (Applause).

*Dr. Miller.* With reference to the shipment of cattle I wish to say a word or two in reply to some statements that were made by the gentleman who read the paper on the transportation of cattle. As has already been said by Professor Michener, if they have in Boston illy-ventilated and badly disinfected cars, that condition certainly does not apply to other parts of the United States. I speak for the port of Philadelphia, positively ; and I can say as much for the port of New York, because I have been there very often, and can speak of Baltimore as well. In reference to our yards in Philadelphia, I can say that they are paved and drained and covered. Cattle upon their arrival there are put in those sheds, and we have a particular part of the yard set aside especially for export cattle. They are cared for just the same as they were prior to the time they left the barns from which they came. There are independent troughs in each stall, and separate feeding troughs as well as watering troughs. We know about the time they are to arrive, and there is hay put in the boxes, so that they are not disturbed after their arrival by the putting in of feed. Hay is not given to them in such quantities as to allow them to overfeed themselves. They are then watered and after a while given a little more feed. The cattle are in very good condition when they arrive there. The cars in which they are shipped are the best the railroad can get at the present time. I have a statement made by Martin, Fuller & Co., to me. They are the largest shippers of stock. They told me that during the last year their percentage of loss between Chicago, the principal shipping point, and Philadelphia, had been less than one per cent. Since I have been at that point examining export cattle, I have had but two animals to die upon the road between Chicago and Philadelphia. One of those died from getting down in the car and being overheated, and the other had to be killed at Pittsburg on account of having his leg broken between Chicago and Pittsburg. There has not been the loss of a single animal that left Chicago between that point and Philadelphia, except these two cases I state. That shows you that they must be carefully started from Chicago and in good condition when they come. There is not a car arriving there that is not thorougly disinfected before it leaves. The cars are cleansed and disinfected before I allow them to leave port.

Dr. Bryden also stated that there were too many cattle crowded in the cars. I do not know what they do in Boston, but in Philadelphia we do not do that. The cattle are loaded so that they can stand comfortably and have sufficient room at the same time. The cars are loaded with from fifteen to twenty-one head, and if the cattle are very large, only sixteen are allowed to be put in a car. They are all unloaded in Pittsburg, fed and watered and re-loaded in the same cars before being shipped from there, and in the same cars in which they leave Chicago they arrive in Philadelphia. After unloading in Philadelpia, the cattle are detained twenty-four hours, and in very few instances, as a rule, have they been shipped in the steamer before the expiration of the twenty-four hours rest. Where they have been shipped prior to the twenty-four hours rest it has been only by the consent of the Secretary. For instance, a train has been delayed, and they did not want to detain the steamer. In one case I remember that the cattle were delayed all night, and reached Philadelphia too late to rest the allotted time of twenty-four hours, but we allowed them to be loaded on the boat. Otherwise the boat would have been detained twenty-four hours longer than the time set for sailing, and that would have been a great expense to the shippers. I will also add that the cattle are loaded into the boat under my personal supervision.

The Doctor also stated that the space allowed the cattle is not sufficient. The two feet six inches on the Spar Deck will not do. I take exception to that, and you will see the soundness of my exception. To everybody who has had anything to do with the shipping of cattle it is plain that if you put a certain number of cattle in a car, leaving just enough room for them to stand comfortably together they will ride more easily than if they were allowed too much space. If too much space is allowed they will oscillate and bruise each other with the motion of the boat or car. In those boats they are loaded in stalls and four cattle are put in each stall, allowing below deck two feet eight inches, and on the upper deck only two feet six inches. But the regulations will require the new boats to allow 2 feet 6, and we are confined to the old regulations in the old boats, because to make a change now would entail the entire refitting of the old boats, which would be a great expense to the shipper. On account of the rocking in the boat it is necessary that the cattle stand on the upper deck a little closer than they do below deck. There is also below deck another and different situation from the upper deck. There are other influences working, such as the cattle below not having the ventilation that those above have and they need a little more circulation than the cattle do upon the Spar Deck. If I had the regulation of the matter I would give more room below and give a space of two feet eight inches above. It has been the wish to put the small cattle in as great numbers as possible on the upper deck, and I have to confine myself to that regulation as much as I can. Those cars coming with twenty and twenty-one head I load on the Spar Deck because they do not take quite so much room as the larger cattle do. So far as the port of Philadelphia is concerned I believe that the regulations required by the Agricultural Department are thoroughly carried out. I follow them as far as it is in my power to do, and certainly there has been no cruelty to animals at that place, and I have been careful not to allow any hanging of the cattle in that port. Nearly all the men that go from Philadelphia with the cattle are men who have been going from Philadelphia to London, Glasgow and Liverpool and back ever since we have been exporting cattle from here to there. Of course they take a certain number of stiffs but they go with the regular men who are thoroughly experienced cattle men and are under supervision. The cattle as a rule arrive in London in good condition and with but few lost. When the losses occur they are from storms at sea where they have been washed from the upper deck.

*Dr. Faville.* I only wish to say a word or two in this matter. I can simply corroborate what Dr. Miller said with reference to the port of Philadelphia and say the same thing in reference to our port at Baltimore. In Baltimore our yards are clean throughout and each yard thoroughly drained and all water is carried off. The yards in Baltimore are dryer and cleaner I dare say than nine-tenths of the farm-yards throughout the United States. Furthermore, in Baltimore they are building an addition to the yards at an expense of $400,000. $400,000 will buy considerable lumber and will give a good sized yard. As far as the shipping of cattle in cars is concerned, not one car out of fifty that comes of Baltimore but is one or the other of the so-called palace animal cars.

Our cattle there are loaded not heavier than 15 to 16 and sometimes 17 cattle to a car. Out of something over 58,000 head, we have exported from the port of Baltimore there have been only 3 or 4 cattle that have been so hurt in transportation as not to be fit to ship. That is a very small percentage. Some of the cattle are not detained in the yard the full 24 hours, because they have not had a long run. This is cattle coming from the vicinity of North Virginia, and Pittsburg. The majority of the cattle remain there nearer 36 hours than 24. So far as cruelty of cattle on board ship is concerned, the scales ought to be a pretty good index, and it is a fact easily proved that very many cargoes gain in transit across the water and weigh more when they reach London or Liverpool than they weighed at the time they left our port.

*Dr. Miller.* There is one point I had forgotten to refer to. The statement is also made by Dr. Bryden that the cattle are too long in transit across the country. After the cattle are loaded in East St. Louis, or in Chicago, or in Pittsburg, according to the regulation of the Department the Inspector at the point of loading shall notify me at Philadelphia for cattle destined to Philadelphia of their having been shipped. He must send me the list containing the number of the car, the railroad the car belongs to, etc. That list comes by mail. It is not sent by the train as the manifest is. I can say that in about seven-tenths of the cases the cattle will be in Philadelphia and I will get the manifest from the railroad company before I get that order through the mail, notwithstanding the cattle will have been unloaded and fed in Pittsburg. That shows whether they come through quickly or not.

*Dr. Faville.* I have had the same experience two or three times.

*Dr. Bryden.* The method pursued at present in carrying out the shipment abroad is not one which will bring better results, so far as freedom for hardship is concerned, as was obtained under the old system when there were no regulations for the shipment of animals. The fact is that they have attacked the wrong end. The Inland transportation has most to do with the condition in which the cattle are received abroad. You go to work and embarrass the ships, I say that is wrong, I simply make these remarks as showing that somebody has been doing an inconsistent thing.

*Dr. Miller.* The gentleman makes a statement here which if true in Boston seems very strange. He said the United States Government had four steamship lines to do certain things. Now it is a fact that all these arrangements have been brought about after having conferences with the agents of several steamship lines themselves, as well as of the agents of railroad companies. I have this directly from the Department. They inform me that these arrangements were made after a thorough consultation with several steamship companies' agents, and the agents of the railroad companies, so that if the arrangements are not satisfactory to the transportating lines they have themselves to blame. He made the statement that distillery cattle had been shipped from Canada and elsewhere, and from twenty to twenty-five died on the wharves at a time in Boston. I do not know what kind of distillery they feed up there. But it is not so down our way. Cattle were shipped through from Kentucky to Philadelphia during the hot weather last month with the single loss of but one animal; 639 head came all the way from Covington, Kentucky, and were loaded on the boat at Philadelphia within twenty-four hours after their arrival at that point, and every one of those animals were received from the distillery at that place, and but one died. If all the grievances exist that the gentleman complains of he is guilty himself of not stopping them, as a simple complaint to the United States Prosecuting Officer would have brought the guilty party to justice. If he knew of these abuses he should have gone to the authorities and complained that the railroad people were wantonly killing the cattle. He is guilty himself if he did not make complaint. He should, if a citizen of the United States, have made complaint. He should have made complaint that the railroads and transportation companies were not complying with the law, and I do not believe anybody in the country would be more ready and anxious to punish the offender than the Secretary of the Agricultural Deparment.

*Dr. Bryden.* Instead of changing the spaces on the ship I should say it would be better to change the size of the stanchions. Do you mean to tell me for a moment that a great distillery is as good a place to ship cattle from, as a stock-yard is? The chances for doing wrong in a stock-yard are entirely obliterated. I say further that instead of changing your ships, you ought to see that no animal of a certain grade is allowed to be shipped. That is the point. Make this a respectable business, and ship no cattle except it comes up to a certain grade.

*Dr. Faville.* In conversation with the manager of the steamship line running out of Baltimore within the last week or two he very emphatically said that if they could do away with the regulations established by the Agricultural Department and which we are enforcing, that they would be most sincerely and emphatically opposed to it. In other words, they prefer to have the regulations enforced to the letter on all of their boats rather than go back to the old way of go as you please.

*Dr. McLean,* I move that the discussion close.

The motion was agreed to.

*The President.* You now have before you the discussion of the paper of Dr. Williams on "Rachitis."

*Dr. Miller.* I wish to say that I consider it one of the best papers on that subject I ever had the pleasure of listening to. I was interested in the case of a colt which has similar to the case cited. I had under my treatment a similar case. The cases were almost identical. The colt belonged to Dr. Agnew. There was the enlargement of the bones and everything else. Before the colt died the limbs softened so that the legs separated at the joints. The doctor would not allow the colt to be killed, as he wanted it kept in the interest of science simply to see how long the colt would live in that condition. The colt lived thirty-six hours after the legs began to drop off. The bones were placed in a barrel and there are many that have already crumbled and gone to pieces.

*Dr. Faust.* The hour is too late to give this paper a full discussion. This is the first time to my knowledge that I ever heard in the English literature that these three diseases were identical in their pathology. I refer to "Rachitis," "Osteo Porosis" and "Osteo Malasia." My heart jumped for joy when I found somebody to think as I had thought, and as I have found universally accepted in the best German Works.

*Dr. Berns.* I was very much interested in it, and I think a good thorough discussion on the subject would be not only interesting but beneficial to all. But as the hour is getting so late I would prefer that the discussion lay over until our next meeting. The paper was a lengthy one. We need more time to study it carefully, and such study will conduce to a more intelligent discussion when the matter comes up again. I hope the discussion will be laid over until the next meeting.

*The President.* If there is no objection it will go over. I thing the suggestion is a good one. I agree with what Dr. Faust has said. I am glad that some one has made public the identity of the three diseases. The hour is getting late, and I would like to know if there are any suggestions in regard td the paper I have read. If there is no further discussion I will, as President, announce the Board of Censors for the following year.

| | |
|---|---|
| Dr. J. T. Winchester, | Dr. William Dougerthy, |
| Dr. R. A. McLean, | Dr. A. Liautard, |
| Dr. T. B. Rayner, | Dr. J. H. Stickney, |

Dr. Olof Schwartzkopff.

The Committees will be appointed and announced later. We will now hear the report of the Treasurer, which was read.

*The Secretary.* I move that the Association extend thanks to the Washington Local Committee and the Baltimore Committee, and the proprietor of Willard's Hotel for the courtesies extended and the reception on the whole given us by the entire community,

The motion was agreed to unanimously.

There upon (five o'clock and thirty minutes P. M.), the Convention adjourned *sine die.*

